# The ChlD subunit links the motor and porphyrin binding subunits of magnesium chelatase

**DOI:** 10.1042/BCJ20190095

**Published:** 2019-07-02

**Authors:** David A. Farmer, Amanda A. Brindley, Andrew Hitchcock, Philip J. Jackson, Bethany Johnson, Mark J. Dickman, C. Neil Hunter, James D. Reid, Nathan B. P. Adams

**Affiliations:** 1 Department of Molecular Biology and Biotechnology, The University of Sheffield, Sheffield S10 2TN, U.K.; 2 Department of Chemical and Biological Engineering, ChELSI Institute, The University of Sheffield, Sheffield S1 3JD, U.K.; 3 Department of Chemistry, The University of Sheffield, Sheffield S3 7HF, U.K.

**Keywords:** AAA proteins, integrins, protein–protein interactions, tetrapyrroles

## Abstract

Magnesium chelatase initiates chlorophyll biosynthesis, catalysing the MgATP^2−^-dependent insertion of a Mg^2+^ ion into protoporphyrin IX. The catalytic core of this large enzyme complex consists of three subunits: Bch/ChlI, Bch/ChlD and Bch/ChlH (in bacteriochlorophyll and chlorophyll producing species, respectively). The D and I subunits are members of the AAA^+^ (ATPases associated with various cellular activities) superfamily of enzymes, and they form a complex that binds to H, the site of metal ion insertion. In order to investigate the physical coupling between ChlID and ChlH *in vivo* and *in vitro*, ChlD was FLAG-tagged in the cyanobacterium *Synechocystis* sp. PCC 6803 and co-immunoprecipitation experiments showed interactions with both ChlI and ChlH. Co-production of recombinant ChlD and ChlH in *Escherichia coli* yielded a ChlDH complex. Quantitative analysis using microscale thermophoresis showed magnesium-dependent binding (*K*_d_ 331 ± 58 nM) between ChlD and H. The physical basis for a ChlD–H interaction was investigated using chemical cross-linking coupled with mass spectrometry (XL–MS), together with modifications that either truncate ChlD or modify single residues. We found that the C-terminal integrin I domain of ChlD governs association with ChlH, the Mg^2+^ dependence of which also mediates the cooperative response of the *Synechocystis* chelatase to magnesium. The interaction site between the AAA^+^ motor and the chelatase domain of magnesium chelatase will be essential for understanding how free energy from the hydrolysis of ATP on the AAA^+^ ChlI subunit is transmitted via the bridging subunit ChlD to the active site on ChlH.

## Introduction

The first committed step in the biosynthesis of chlorophyll is catalysed by magnesium chelatase (MgCH; E.C.6.6.1.1), a large, multisubunit enzyme which catalyses the insertion of a Mg^2+^ ion into protoporphyrin IX in a Mg^2+^ and MgATP^2−^-dependent manner. In photosynthetic organisms, MgCH, along with ferrochelatase, lies at the branch point of the chlorophyll and haem biosynthesis pathways. While ferrochelatase is a relatively simple, single subunit enzyme, MgCH is a large complex, with a minimal catalytic requirement of three subunits: in oxygenic phototrophs, these subunits are named ChlI, ChlD and ChlH [1–3]. The insertion of a Mg^2+^ ion into protoporphyrin is energetically unfavourable [4,5], and proceeds only via coupling to an ATPase motor subunit, ChlI. The ChlI and ChlD subunits are members of the AAA^+^ (ATPases associated with various cellular activities) superfamily of enzymes [6]. While ATPase activity has been observed and measured for the ChlI subunit [7–10], no ATPase activity has been observed for ChlD. ChlI hydrolyses MgATP^2−^ to provide the considerable power required to drive Mg^2+^ insertion [5,11]. ChlD appears to act as an allosteric regulator in response to MgATP^2−^ concentrations [12], and the C-terminal domain of ChlD regulates the cooperative response to Mg^2+^ concentrations in the *Synechocystis* sp. PCC 6803 (hereafter *Synechocystis*) MgCH, although not in the *Thermosynechococcus elongatus* enzyme [13]. Previous work has shown the importance of a fully intact ChlD (or bacterial homologue, BchD) protein for maintaining chelatase activity [11,13,14], although recent work published by Luo et al. [15] suggests only the N-terminal AAA^+^ and polyproline linker domains are required for activity.

The ChlH subunit is a large protein (∼150 kDa) that binds protoporphyrin IX [16,17]; recent crystallographic evidence has provided clues to the porphyrin binding site [18]. Mg^2+^ and MgATP^2−^ are required for the association of ChlI and ChlD [12,19], mediated by the AAA^+^ N-terminal domains of each subunit, which share ∼40% sequence identity. Additionally, ChlD has an extended linker region rich in acidic residues and a C-terminal integrin I domain, which is also known as von Willebrand factor A (VWFA), and these domains are usually associated with protein interactions involving cell adhesion [20]. It is generally assumed that ChlID associates with ChlH to form an active ChlIDH complex, but the number and spatial arrangement of subunits required to catalyse the chelatase reaction are currently unknown.

While there have been an extensive steady state [2,4,5,12,13,21–26] and transient [27] kinetic analyses of MgCH, there is no settled view on the organisation of subunits and the protein–protein interactions representing the minimal catalytic unit. In addition, it is not known how energy from ATP hydrolysis is transmitted to the catalytic site on ChlH. Here we show that ChlD and ChlH form a complex both *in vivo* and *in vitro*. Using chemical cross-linking coupled with analysis by mass spectrometry (XL–MS) and further biophysical analysis, we propose the C-terminal integrin I domain of ChlD forms the majority of interactions with ChlH, with some interaction mediated by the AAA^+^ domain of ChlD. This knowledge provides the basis for the organisation of the ChlHID magnesium chelatase complex and for exploring how chemomechanical coupling, produced from the hydrolysis of ATP on the AAA^+^ ChlI subunit, is moved to the metal ion insertion site on ChlH via the bridging subunit ChlD.

## Materials and methods

### Growth of *Synechocystis*

Strains used in this study are detailed in Supplementary Table S1. *Synechocystis* strains were grown at 30°C in BG11 medium [28] buffered with 10 mM *N*-Tris(hydroxymethyl)methyl-2-aminoethanesulfonic acid (TES)-KOH pH 8.2 (BG11-TES). Starter cultures were grown in a rotary shaker (150 rpm) at a constant illumination of 40 µmol photons m^−2^ s^−1^. For purification of FLAG-tagged proteins 8 l cultures of *Synechocystis* were grown with vigorous bubbling with sterile air at a constant illumination of 150 µmol photons m^−2^ s^−1^. Growth was monitored as the optical density at 750 nm (OD_750_). For growth on solid medium, BG11-TES was supplemented with 1.5% (w/v) agar, 0.3% (w/v) sodium thiosulphate and appropriate antibiotics and plates were incubated at 30°C with 40 µmol photons m^−2^ s^−1^ constant illumination.

### Construction of *Synechocystis* strains

Details of primers used in this study are provided in Supplementary Table S2. To generate strain FLAG-ChlD, the *chlD* gene (slr1777) was amplified from *Synechocystis* DNA with flanking *Not*I and *Bgl*II sites using Q5 High Fidelity DNA polymerase (NEB, U.K.) and primer pair AH282/AH283. This fragment was cloned into the *Not*I and *Bgl*II sites of pPD-NFLAG [29] such that it was in frame with an N-terminal 3хFLAG tag. The resulting plasmid was introduced into wild-type *Synechocystis* by natural transformation. Transformants were selected on BG11 agar containing 7.5 µg ml^−1^ kanamycin and genome copies were segregated by a sequential doubling of the antibiotic concentration to 60 µg ml^−1^. Segregation at the *psbAII* locus was confirmed by PCR using primers AH47/AH48 (Supplementary Figure S1A) and the sequence of the FLAG-ChlD encoding gene was verified following amplification from transformant genomic DNA (GATC Biotech, Germany).

To delete the native *chlD* (slr1777) from strain FLAG-ChlD, a linear DNA construct was generated to replace 1943 bp of the 2031 bp gene with the zeocin resistance cassette (zeoR). Fragments of approximately 500 bp flanking the *chlD* gene were generated by PCR using *Synechocystis* genomic DNA as a template with primers AH434/AH435 and AH436/AH437, and zeoR was amplified from pZEO using primers Zeo-F and Zeo-R [30]. These three fragments were purified and used in an overlap extension (OLE)-PCR with primers AH434/AH437, resulting in a ∼2.0 kb product, which was introduced into the FLAG-ChlD strain as above. Transformants were selected on BG11 agar containing 2.5 µg ml^−1^ zeocin and genome copies were segregated by a sequential doubling of the antibiotic concentration to 20 µg ml^−1^. Absence of the native *chlD* from the resulting FLAG-ChlD Δ*chlD* strain was confirmed by PCR with primers AH434/AH437 and AH434/AH438 (Supplementary Figure S1B,C).

### Co-production of StrepII-ChlH and ChlD in *E. coli*

The region between the start codon and the end of the hexaHis-tag in pET14bSynChlH [26] was replaced by the region between the start codon and the end of the StrepII-tag (WSHPQFEK) in pET52b (Novagen) by replacing the 92 bp between the *Xba*I and *Nde*I restriction sites with a 95 bp synthetic fragment (Integrated DNA Technologies), generating plasmid pET14bStrepII-SynChlH. The QuikChange II kit (Agilent) was used to introduce an *Spe*I site downstream of the stop codon of *chlH* in pET14bStrepII-SynChlH. The QuikChange II kit was also used to engineer an *Spe*I site downstream of the *Bam*HI site in both pET9aHis-ChlD [12] and pET3a (Novagen), and the *chlD* gene was excised from pET9aHis-ChlD with *Nde*I and *Spe*I and sub-cloned into the same sites of the modified pET3a, producing plasmid pET3aSynChlD. We then used the ‘Link and Lock’ method [31] to insert *chlD* with its own RBS downstream of the stop codon of the tagged *chlH* gene in pET14bStrepII-SynChlH. The pET3aSynChlD plasmid was digested with *Xba*I and *Hin*dIII and the released fragment was ligated into *Spe*I-*Hin*dIII cut pET14bStrepII-SynChlH, creating pET14bStrepII-SynChlH-SynChlD for co-production of N-terminally StrepII-tagged ChlH and untagged ChlD. All plasmids were verified by automated DNA sequencing (GATC, Eurofins Genomics).

### Calculation of chlorophyll concentrations in *Synechocystis* strains

Cell number was normalised to OD_750_ and the equivalent of OD_750_ = 1 was mixed directly with a total volume of 1.5 ml methanol and incubated at room temperature for 10 min with gentle shaking. Cell debris was cleared via centrifugation at 16 000×***g*** for 10 min and the concentration of chlorophyll was calculated using the extinction coefficient 71 mM^−1^ cm^−1^ at 665 nm [32].

### *In vivo* FLAG-ChlD immunoprecipitations

*Synechocystis* strain FLAG-ChlD was grown to an OD_750_ of 0.9. Cells were pelleted and resuspended in buffer A (25 mM sodium phosphate, 50 mM NaCl, 10 mM MgCl_2_, 10% (w/v) glycerol pH 7.4 and EDTA-free protease inhibitors). Cells were mixed with an equal volume of 0.1 mm glass beads and broken in a Mini-Beadbeater-16 (BioSpec) (50 s beating, 2 min cooling on ice, 10 cycles). Soluble proteins and membranes were separated by centrifugation (65 000×***g***, 30 min, 4°C). The membrane fraction was washed with an excess of buffer A, then resuspended in buffer A with 1.5% (w/v) dodecyl-*β*-maltoside (β-DDM, Applichem) and solubilised for 2 h at 4°C. The solubilised membranes were separated from insoluble material by centrifugation (65 000×***g***, 30 min, 4°C).

FLAG-ChlD and associated proteins were purified from the soluble and membrane fractions using a 200 µl anti-FLAG-M2 agarose column (Sigma–Aldrich) equilibrated in buffer A (+0.25% (w/v) *β*-DDM for membrane fraction). FLAG-ChlD was eluted with 500 µl of buffer A (+0.25% β-DDM for membrane fraction) containing 150 µg/ml 3xFLAG peptide (Sigma–Aldrich).

The FLAG-ChlD co-immunoprecipitation complex was seperated by denaturing gel electrophoresis using NuPage 12% Bis–Tris gels (Life Technologies) and proteins were transferred onto a polyvinylidine fluoride membrane for immunodetection. Immunoblotting and detection were performed as described previously [29].

### Purification of StrepII tagged ChlH

The pET14bStrepII-SynChlH-SynChlD was transformed into *E. coli* Rosetta^™^(DE3)pLysS and grown in 2× YT medium to an OD_600_ of 0.6; protein overproduction was induced with the addition of 0.4 mM IPTG and flasks incubated for a further 12 h at 16°C. Cells were suspended in binding buffer (100 mM Tris HCl, 1 mM EDTA, pH 8) and lysed by sonication (6 × 30 s with 30 s breaks), and cell debris cleared by centrifugation (15 m, 53 000×***g***). The supernatant was applied to a StrepTrap HP column (GE Healthcare) pre-equilibrated in binding buffer and unbound proteins removed with 15 column volumes of binding buffer. ChlH-StrepII was eluted with binding buffer supplemented with 2.5 mM desthiobiotin. Western blot analysis was performed in essentially the same manner as described above.

For the control experiment with non-tagged ChlD cells were grown and proteins purified in an identical fashion.

### Purification of chelatase subunits

Production and purification of MgCH subunits were performed as described previously [5,12,13]. Point mutants were produced using the QuikChange II site-directed mutagenesis kit (Agilent, U.K.) with the primers listed in Supplementary Table S3 and verified by sequencing (GATC Biotech).

### Thermophoresis

ChlH or ChlD was labelled with NHS-NT647 dye (NanoTemper Technologies, Munich, Germany) according to the manufacturer's instructions before exchange into microscale thermophoresis (MST) buffer (50 mM Tris/NaOH, 0.2% pluronic F-127, with or without 10 mM MgCl, pH 7.8). MST experiments were performed in triplicate, with 20 nM labelled chelatase subunit titrated with a serial dilution of a partner protein. Samples were loaded into premium capillaries (NanoTemper Technologies) and illuminated with 20% RED LED power, with thermophoresis induced with 40% IR laser power. Thermophoresis data were analysed as previously described [11].

### Chemical cross-linking of proteins

Purified ChlH was desalted into activation buffer (100 mM MES, 0.5 M NaCl, pH 6) using a Zeba Spin column according to the manufacturer's instructions. 1 mg ml^−1^ of ChlH in 100 µl activation buffer was incubated with 2 mM 1-ethyl-3-[3-dimethylaminopropyl]carbodiimide hydrochloride (EDC, Thermo Fisher) and 5 mM *N*-hydroxysuccinimide (NHS, Sigma–Aldrich) at room temperature for 15 min and then buffer exchanged into PBS. 1 mg ml^−1^ activated ChlH was mixed with 1 mg ml^−1^ ChlD and incubated at 34°C for 2 h prior to flash-freezing samples.

Cross-linked samples were analysed by SDS–PAGE using NuPAGE 3–8% Tris–acetate gels in denaturing buffer (Tris–glycine-SDS), and cross-linked protein detected by immunoblotting for *Synechocystis* ChlD and ChlH, using the methods reported above.

### In-gel digestion and mass spectrometry analysis

Bands corresponding to putative ChlH–ChlD complexes were excised and subjected to in-gel digestion with trypsin according to [33]. The peptide extracts were dried in a vacuum centrifuge, redissolved in 10 µl 0.1% (v/v) TFA, 3% (v/v) acetonitrile and 2 µl analysed by nano-flow liquid chromatography (Ultimate 3000 RSLCnano system, Thermo Scientific) coupled to a mass spectrometer (Q Exactive HF, Thermo Scientific) according to [34]. The mass spectra were processed with Byonic v. 2.9.38 (Protein Metrics, CA, U.S.A.) with parameters set to default except that carbamidomethyl-Cys and Met oxidation were specified as fixed and variable (common) modifications, respectively. Protein identifications were made by searching the Cyanobase *Synechocystis* sp. PCC 6803 proteomic database (http://www.uniprot.org/uniprot/?query=organism:1111708) using the Byonic search engine (Protein Metrics). The identification of cross-linked peptides was enabled by manually creating a rule for EDC with the following syntax: ‘EDC/-18.010565 @ K,D,E | xlink’. Cross-linked peptides were identified in Byonic using a two-dimensional posterior error probability (2-D PEP) <0.01 as a threshold. All cross-linked peptides identified in Byonic were manually verified.

### Kinetic assays of magnesium chelatase activity and data analysis

Kinetic assays were performed as described previously [12]. Analysis was conducted in MARS Data Analysis Software Version 1.x R2 (BMG Labtech) and Igor Pro Version 7.0.6.1 (Wavementrics, Lake Oswego, OR).

### Producing a homology model for the integrin I domain of ChlD

Residues 481–672 of the *Synechocystis* enzyme were highlighted by Prosite [35] to belong to the VWFA family. These residues were aligned using HHPRED [36] against sequences with known structures in the PDB. An alignment with hit TEM8 was generated using the HHPRED-TemplateSelection tool and this alignment used to create a model with Modeller (v9.21) [37].

### Differential scanning fluorimetry assays for thermal stability of MIDAS mutants

Each assay contained 5 µM ChlD, 10 mM MgCl_2_, 1× SYPRO Orange and 1× protein buffer to give a final volume of 50 µl. The QPCR machine (Agilent) was programmed to generate a temperature increase of 1°C per cycle per minute. A fluorescence reading was taken each cycle. Melting temperatures (*T*_m_) were assigned at the point where 50% of the protein is unfolded, as determined by fitting melting data to a sigmoid Boltzmann distribution (eqn 3) where *F*_0_ is initial fluorescence, *F*_max_ is maximal fluorescence, *T* is the temperature, *T*_m_ is the melting temperature and *T*_0_ is the initial temperature.

3 $$F=F_{0}+\left(\frac{F_{\max}}{1+{\text{exp}}^{T_{M}-T_{0}/\frac{\delta T}{\delta F}}}\right).$$

### CD spectrometry of MIDAS mutants

Spectra were recorded with a JASCO-810 spectrometer (JASCO, Great Dunmow, U.K.). Protein (0.05 mg ml^−1^ was in a 5 mM sodium phosphate buffer, 1 mM β-mercaptoethanol, pH 7.5. Spectra were recorded from 260 to 200 nm (1 nm steps, 4 s nm^−1^, four accumulations) and background subtracted.

## Results

### *Synechocystis* ChlH and ChlD form a membrane-associated complex *in vivo*

**Figure 1.**
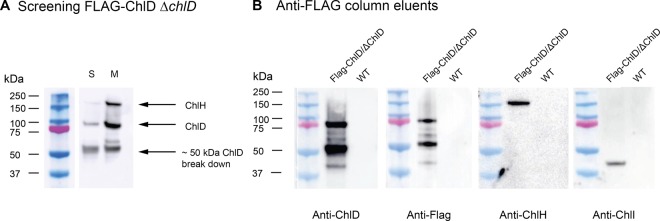
Purification of FLAG-ChlD–ChlH–ChlI complex from *Synechocystis* cells. (**A**) FLAG-ChlD Δ*chlD Synechocystis* soluble and detergent solubilised extracts were applied to an anti-FLAG column to optimise purification conditions. Proteins were separated by 12% Bis–Tris SDS–PAGE, electro-transferred to a PVDF membrane and probed with antibodies for ChlH and ChlD. Lanes: S, soluble fraction elution; M, membrane fraction elution. The ChlD–ChlH complex detected in both soluble and membrane fractions, with larger amounts of both proteins present in the membrane fraction. (**B**) Probing with antibodies for each chelatase subunit in FLAG-ChlD Δ*chlD Synechocystis* and wild-type (WT) *Synechocystis* membrane fractions eluted from a FLAG column revealed that the three constituent proteins of MgCH can all be co-purified from solubilised membranes when there is a N-terminal FLAG tag on ChlD.

To investigate the *in vivo* association of ChlD with the other chelatase subunits, we constructed a *Synechocystis* strain producing ChlD with a 3xFLAG-tag encoded at the N-terminus expressed under the control of the *psbAII* promoter (FLAG-ChlD), and then deleted the native *chlD* gene from this strain (FLAG-ChlD Δ*chlD*). (Supplementary Figure S1 and Supplementary Table S1). The cellular concentrations of chlorophyll in wild-type, FLAG-ChlD and FLAG-ChlD Δ*chlD* were similar (data not shown). Soluble and membrane fractions were prepared from photoautotrophically grown FLAG-ChlD strains, then membranes were solubilised in a buffer containing 1.5% (w/v) β-DDM and the extracts applied to an anti-FLAG column. After extensive washing, eluted material was separated by SDS–PAGE and specific antibodies for ChlH and ChlD were used to determine the presence of ChlH and ChlD (Figure 1A). In both soluble and membrane fractions, ChlH co-purifies with FLAG-ChlD (Figure 1A, lanes S and M, respectively).

Despite not being integral membrane proteins, both ChlD and ChlH proteins are present in higher concentrations in the membrane fraction than in the soluble fraction (compare lanes S and M, Figure 1A). The ChlD protein appears to be unstable both *in vitro* and *in vivo*, and a ∼50 kDa breakdown product is evident when blotting for either ChlD or the FLAG tag, indicating the 50 kDa fragment is from the N-terminal region of the protein. We further analysed the membrane fraction, using immunoblots to probe for each obligate subunit of MgCH (ChlI, ChlD and ChlH) in fractions of membranes from WT and FLAG-ChlD Δ*chlD* strains (Figure 1B). This analysis revealed that the entire chelatase complex can be purified from the membrane fraction using the FLAG-tagged ChlD protein. No chelatase subunits bound to the anti-FLAG column in the absence of the FLAG tag on ChlD (see WT lanes, Figure 1B). We note that when purified *in vitro* MgCH is a soluble protein complex, and does not require detergents for purification or activity, and indeed the addition of detergent decreases the *in vitro* activity of MgCH.

### A ChlH–ChlD complex can be co-purified when the subunits are recombinantly produced in *E. coli*

**Figure 2.**
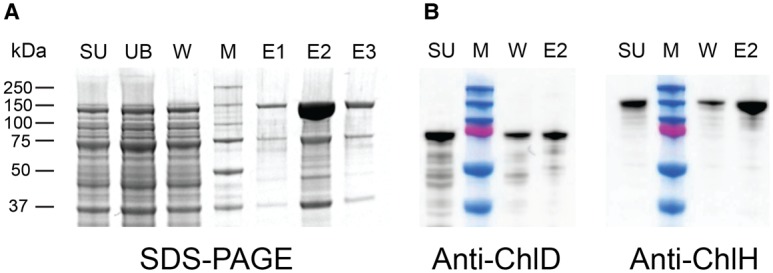
Copurifying recombinant ChlD and ChlH. (**A**) *Synechocystis* StrepII-ChlH was co-produced with non-tagged ChlD and purified on a StrepTrap HP column. Lanes: SU, cell supernatant; UB, unbound fraction; M, molecular mass markers as indicated on the right-hand side; W, Binding buffer wash, Elutions (E1–3): Binding buffer with 2.5 mM biotin. (**B**) Western blot analysis of StrepII-ChlH and non-tagged ChlD fractions from (**A**), showing the presence of ChlD in both the supernatant and elution fraction.

Genes encoding an N-terminal StrepII-tagged ChlH and untagged ChlD from *Synechocystis* were co-expressed in *E. coli*. Proteins were purified by StrepTactin affinity chromatography, using low salt buffers to retain subunit interactions. Analysis by SDS–PAGE showed bands at 150 and 75 kDa corresponding to the molecular masses of ChlH and ChlD, respectively (Figure 2A).Western blot analysis of the supernatant and major elution fraction (E2) confirmed that ChlD co-purified with tagged ChlH (Figure 2B). A control experiment where only non-tagged ChlD was applied to the StrepTactin Sepharose did not show any significant quantity of ChlD binding to or eluting from the column (Supplementary Figure S2).

### Quantitative analysis of binding between ChlD and ChlH

**Figure 3.**
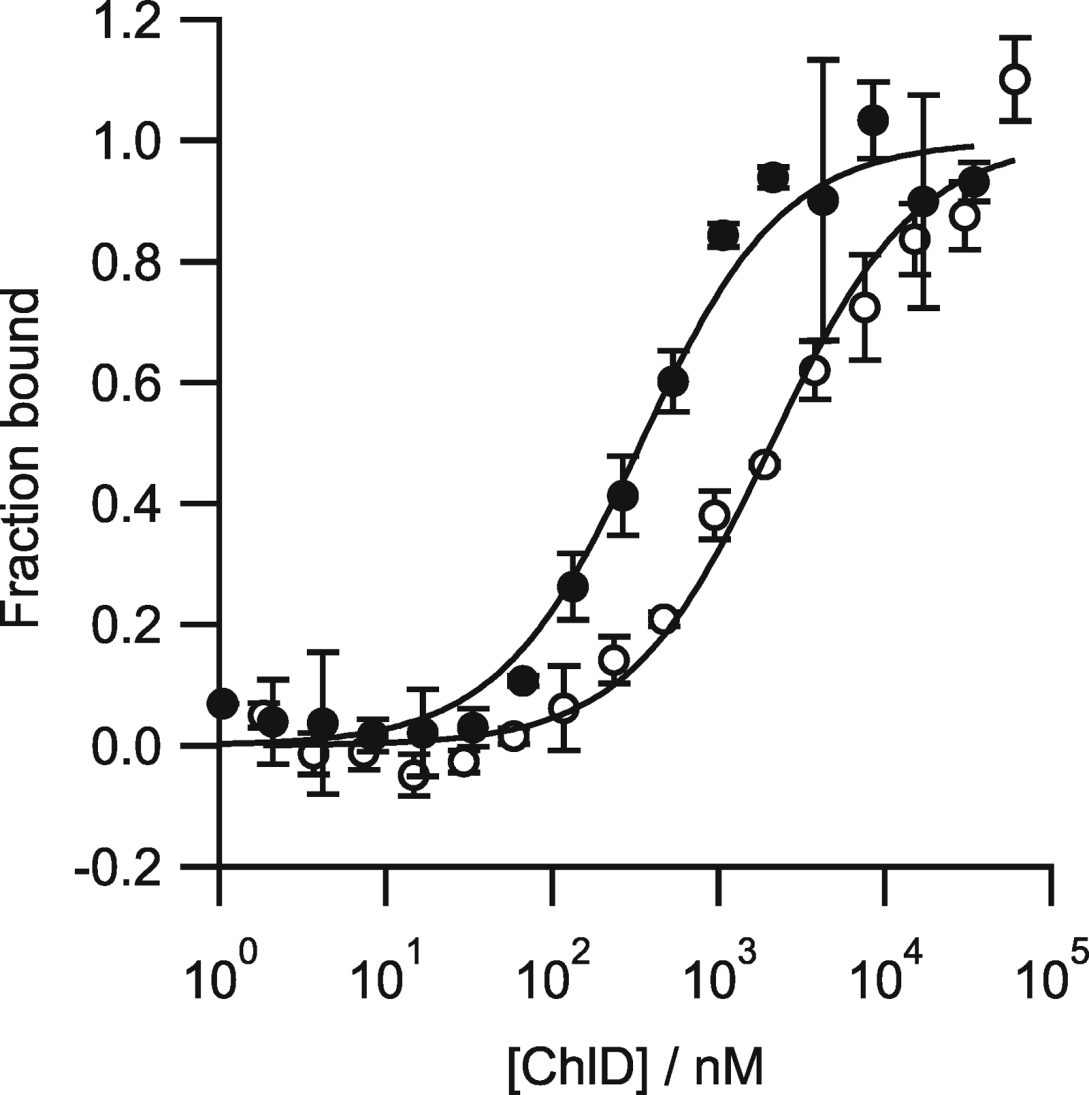
Quantifying the assembly of the ChlD–ChlH complex. (**A**) The presence of magnesium increases the strength of the ChlD–ChlH interaction. Thermophoresis was performed where ChlD was titrated into 20 nM labelled ChlH with or without 10 mM Mg^2+^ in 50 mM Tris/NaOH, 0.2% Pluronic-F127, pH 7.8. The interaction shows a higher affinity at 10 mM Mg^2+^ (filled circles) compared with Mg^2+^ free (open circles). Each data point represents the average of three independent biological repeats, with the standard deviation represented by error bars. Fitting of the resultant binding isotherms revealed *K*_d_ values of 331.99 ± 58 nM and 2088.9 ± 387 nM for Mg^2+^ present and Mg^2+^ free, respectively.

Purified recombinant ChlH and ChlD proteins from *Synechocystis* were produced separately in *E. coli*, then MST was used to determine the binding affinity of ChlD to ChlH in the absence of other subunits or any substrates [11]. ChlD was titrated into a constant concentration of labelled ChlH. Dissociation constants (*K*_d_) values were calculated using a single-site binding model using the MO.Affinity Software (Nanotemper) supplied with the instrument. The interaction between ChlH and ChlD proved to be Mg^2+^-dependent, and a *K*_d_ of 331 ± 58 nM was obtained in the presence of 10 mM Mg^2+^, and increased by an order of magnitude to 2089 ± 387 nM in the absence of Mg^2+^ (Figure 3 and Table 1). Control experiments to determine the assembly state of both ChlH and ChlD within the binding titrations (Supplementary Figure S3) indicate that labelled ChlH is a monomer at the 20 nM concentration in the assay, and that assembly into higher-order species only occurs at concentrations higher than 100 nM. ChlD self-assembles to form a dimer under these experimental conditions, with a calculated *K*_d_ of 296 nM. There is no indication from the ChlD–ChlD titration that any higher-order multimers are being formed within the concentration range of the binding studies presented in this work. This would indicate that the ChlD–ChlH titrations initially represent a monomer of ChlD interacting with ChlH, after which the dimer form of ChlD will likely form the majority of interactions with a monomer of ChlH. Only slight deviations from a single-site binding curve are seen in these data suggesting that more complex assembly modelling is not worthwhile in this case. We emphasise that the measured *K*_d_ values for the ChlH–ChlD and ChlH–ChlD mutant interactions may include components of the ChlD dimerisation and this value should be treated as an empirical description of the interaction between ChlH and all oligomeric states of ChlD.

**Table 1.** Dissociation constant (*K*_d_) values for the ChlD–ChlH interaction of the ChlD mutants

ChlD mutant	Mutation type	Mg^2+^ present *K*_d_/µM	Mg^2+^ free *K*_d_/µM
Wild type		0.331 ± 0.058	2.10 ± 0.387
Truncation A	N-terminal truncation ^(1)^ AAA^+^ domain alone	14.6 ± 2.99	5.93 ± 1.08
Truncation B	N-terminal truncation^(1)^ AAA^+^ and PP domain	10.5 ± 1.9	6.76 ± 1.52
QuinE	Allosteric response to Mg^2+ (2)^	0.339 ± 0.040	0.512 ± 0.161
D487E	MIDAS motif	0.842 ± 0.162	1.489 ± 0.221
S489A	MIDAS motif	1.98 ± 0.52	3.21 ± 0.76
S491A	MIDAS motif	0.147 ± 0.026	0.351 ± 0.097
S554R	MIDAS motif	0.481 ± 0.140	0.988 ± 0.126

*K*_d_ reported to three significant figures, errors reported as ± the estimated standard deviation of the *K*_d_. Each *K*_d_ is determined from three independent biological repeats, errors reported are from non-linear regression. Constructs originally described in (1) Adams et al. [11] and (2) Brindley et al. [13].

### Subunit interactions within the ChlH–ChlD complex

**Figure 4.**
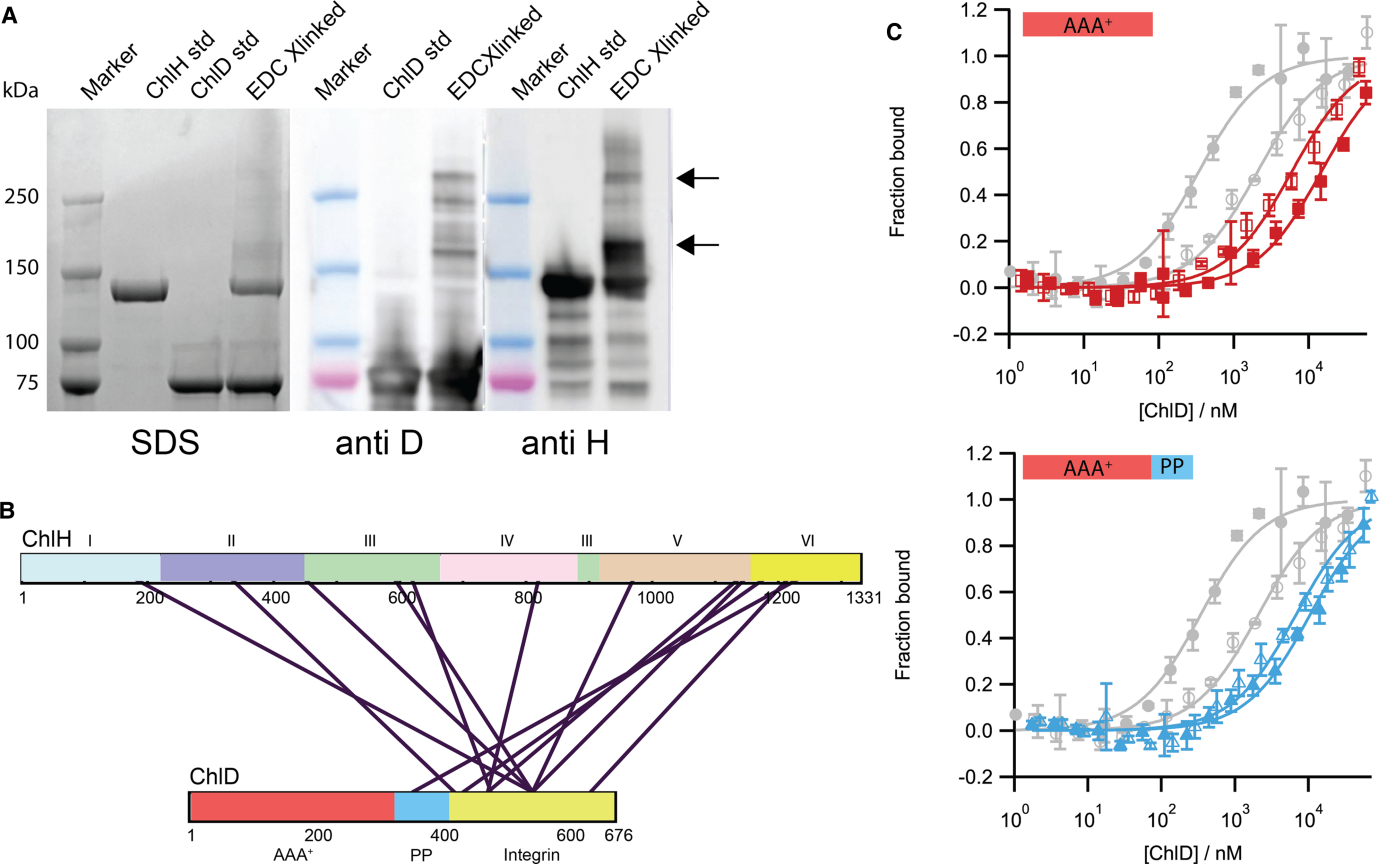
Capturing the ChlD–ChlH complex. (**A**) SDS–PAGE analysis of chemically cross-linked proteins. 1 mg ml^−1^ ChlH was cross-linked with 1 mg ml^−1^ ChlD using EDC (see materials and methods); immunoblotting shows high molecular mass bands containing both ChlH and ChlD (indicated by arrows). Lanes as labelled above. (**B**) Bands indicated by arrows in (**A**) were subjected to in-gel digestion with trypsin and analysed for EDC-cross-linked residues by mass spectrometry; the primary sequence of the ChlH and ChlD proteins are presented as rectangles with residue numbers marked. For ChlH, the six domains are numbered as in Chen et al. [18]. The AAA^+^ (red), acidic polyproline region (PP) (blue) and the C-terminal Integrin domain (yellow) domains of ChlD are indicated. Purple lines denote chemical cross-links between ChlH and ChlD. (**C**) MST assays to measure binding of C-terminal truncations of ChlD to ChlH. Truncations consisted of either the AAA^+^ domain alone (red isotherm) or the AAA^+^ domain with the polyproline region (blue isotherm). Closed markers, 10 mM free Mg^2+^; open markers, no free Mg^2+^. For comparison, WT ChlD isotherms are shown in grey.

Chemical cross-linking was employed to gain an insight into the physical location of the interaction site between ChlH and ChlD. The water-soluble zero length 1-ethyl-3-(3-dimethylaminopropyl)carbodimide hydrochloride (EDC) cross-linker was used, which produced higher molecular mass bands (highlighted with arrows in Figure 4A) in SDS–PAGE and immunoblot analyses. The bands denoted by arrows in Figure 4A were excised from the gel and subjected to in-gel tryptic digestion. Mass spectrometry analysis of the resultant peptides showed that the majority of cross-links appear to occur between the C-terminal integrin I domain of ChlD (shown in yellow in Figure 4B) and the ‘body’ region of ChlH (domains III–VI), although there is also a cross-link in the head region (domain I) of ChlH from residues 150–173 (Figure 4B and Supplementary Table S4 and S5).

### C-terminal truncations of ChlD dramatically weaken the interaction with ChlH

We have previously shown that the N-terminal AAA^+^ domain of ChlD is primarily responsible for the interaction that forms the ChlID motor complex [11]. Cross-linking of ChlH to ChlD (Figure 4B) shows that the majority of interactions with ChlH involve the C-terminal domain of ChlD, but a cross-link with the PP region suggested the N-terminal domain ChlD may also be in close proximity to ChlH. As a test, we used MST to measure the extent to which C-terminal truncations of ChlD retain the capacity to bind to ChlH. These truncation mutants consisted of either the AAA^+^ domain alone (truncation A, Figure 4C, red isotherm), or the AAA^+^ domain with the polyproline region (truncation B, Figure 4C, blue isotherm). The C-terminal truncation mutants show an almost 50-fold higher *K*_d_ compared with wild-type, which weakens even further in the presence of Mg^2+^; this is in stark contrast with the wild-type which shows an order of magnitude tighter binding in the presence of Mg^2+^ (Table 1). Thus, mass spectrometry and MST show that the C-terminal integrin I domain of ChlD is the major determinant of binding to ChlH. In the Luo et al. study [15], where recombinant proteins from rice are used, their results suggest that only the AAA^+^ and linker domains are required for protein activity, results that directly contradict this work, previous studies from our laboratory [11,13] and that of other groups [14]. While it is possible that higher plant enzymes have evolved different interaction sites between the ChlD and ChlH proteins, the proposals of Luo et al. [15] require biophysical characterisation of the protein–protein interactions of the rice proteins.

### The ChlH–ChlD interaction mediates the cooperative response of the *Synechocystis* chelatase to magnesium

The chemical cross-linking and truncation binding studies indicated that the integrin I domain is the primary driver of the interaction between ChlH and ChlD. Previously, we showed that five glutamate residues in the C-terminal region of ChlD (E510, E513, E600, E603 and E605) regulate the cooperative response to Mg^2+^ observed in chelatase assays in the *Synechocystis* enzyme [13]. Binding studies were performed between ChlH and a quintuplet mutant of ChlD with these five glutamate residues altered, to see if the Mg^2+^-dependent decrease in *K*_d_ for the ChlH–ChlD association observed in Figure 3A was related to this cooperativity. The five glutamate residues are replaced with the corresponding residues found in the non-cooperative *T. elongatus* ChlD subunit (E510Q/E513Q/E600T/E603P/E605T). In the presence of Mg^2+^, this mutant (ChlD QuinE) has a *K*_d_ for ChlH similar to the wild-type protein, which is essentially unchanged in the absence of Mg^2+^. This result suggests that Mg^2+^-dependent regulation of the ChlH–ChlD interaction is the source of this cooperativity (Supplementary Figure S4).

### The metal ion-dependent adhesion site is essential for MgCH catalysis

Integrin I domains are often involved in Mg^2+^-dependent protein–protein interactions, mediated by a metal ion-dependent adhesion site (MIDAS) motif (DXSXS where X is any residue) that co-ordinates a Mg^2+^ ion; completion of the magnesium coordination sphere with a ligand from the second protein is presumed to stabilise the protein–protein interaction [38]. Previous studies on the MgCH BchD subunit from bacteriochlorophyll-synthesising *Rhodobacter capsulatus* [14] also provide evidence of the importance of this region in either protein–protein interactions or catalysis.

A homology model for this integrin I domain of ChlD was produced to aid investigation of the MIDAS region. Twenty-one percentage sequence identity was found between residues 481–672 of the *Synechocystis* enzyme and the extracellular domain of human Tumour Endothelial Marker 8 (TEM8, PDB accession code: 3N2N) [39]. The resulting alignment was used with Modeller [37] to generate multiple homology models, with the best one chosen by Modeller's inbuilt DOPE score [40].

**Figure 5.**
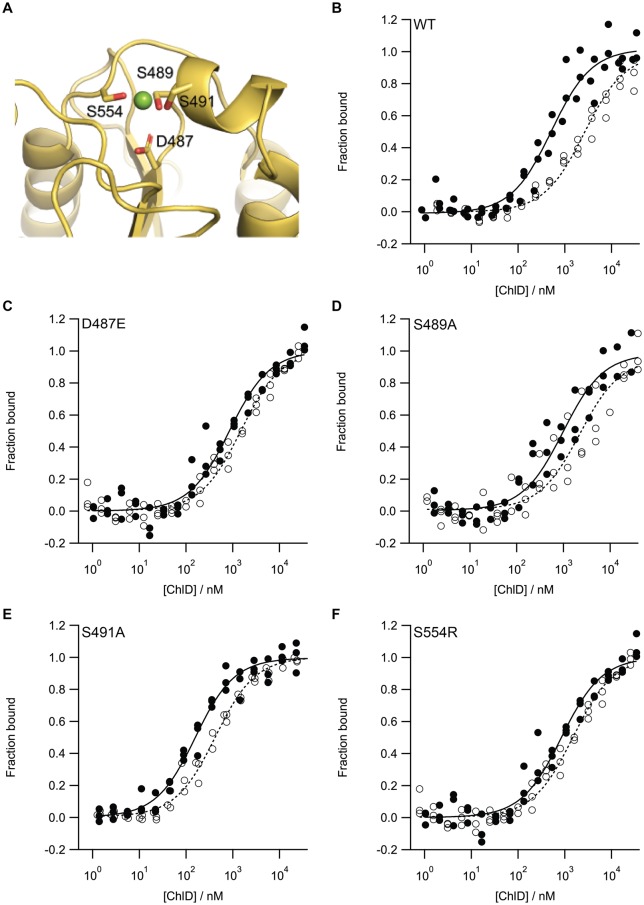
MIDAS mutants of ChlD continue to form a ChlD–ChlH complex. (**A**) Model structure of the MIDAS motif of ChlD and MST binding isotherms of (**B**) WT and (**C**–**F**) MIDAS mutants. In the model structure, ChlD is represented as a cartoon with predicted Mg^2+^ binding residues represented as sticks. MST assays were performed in the presence (closed circles) and absence (open circles) of 10 mM Mg^2+^. MST experiments were performed in triplicate from independent batches of protein. *K*_d_ values are shown in Table 1.

With the aid of the homology model (Figure 5A), a series of point mutations was created to probe the MIDAS motif. Type I mutants (D487E, S489A and S491A; we could only produce low levels of the D487A mutant) perturbed metal binding. The serine mutants lose a putative ligand for Mg^2+^ while the aspartate to glutamate mutation was predicted to add a steric clash with the Mg^2+^. The type II mutant (S554R) introduced an Arg residue, blocking metal binding whilst providing a replacement positive charge. All the recombinant proteins purified as wild type (apart from D487A); their CD spectra were consistent with folded protein, and their melting temperatures were above the standard chelatase assay temperature of 34°C (Supplementary Figure S5 and S6 and Supplementary Table S6).

All the variant proteins were inactive, except for S554R which has a relative rate of ∼5% of that of the wild-type protein (Supplementary Figure S7). To investigate whether the lack of activity was due to a decreased affinity for ChlH, MST was performed on each of the MIDAS mutants in the presence or absence of Mg^2+^ (Figure 5B–F). The *K*_d_ values (Table 1) show that all mutants are able to bind ChlH at least as well as the WT in the absence of Mg^2+^, with ChlD S491A appearing to have a greater affinity for ChlH than WT, especially in Mg^2+^-free conditions. The only active mutant, S554R, has a *K*_d_ value for ChlH roughly similar to that of WT protein. While integrin I domains are generally involved in cell–protein interactions in the case of the S554R mutant, which mimics the charge of a ligated Mg^2+^ ion, the correct ChlD-H association may occur to allow power to be transferred from the ChlID complex. However, the lowered chelatase rate implies that the ChlD–ChlH interaction can still occur if the positive charge associated with the chelated Mg^2+^ in the MIDAS motif is replaced by a positively charged amino acid, although a correctly ligated Mg^2+^ ion in the MIDAS domain is undoubtedly important for full chelatase activity.

## Conclusion

Magnesium chelatase is responsible for the initiation of one of the most important biochemical pathways on Earth, the biosynthesis of (bacterio)chlorophyll. Understanding how this pivotal enzyme transfers the free energy gained from the hydrolysis of MgATP^2−^ at the AAA^+^ motor to the active site where chelation takes place is vital, not only for revealing the mechanism of MgCH but also in our broader understanding of allosteric circuits within the AAA^+^ superfamily of motors. While much work has taken place to understand the individual roles of the subunits within MgCH, only now are we starting to understand the protein–protein interactions that are involved in forming the catalytic ChlIDH complex. Identification of protein–protein interaction domains and motifs will facilitate the elucidation of the stoichiometry of the subunits within the active complex, the allosteric circuits which regulate the movement of chemomechanical motion through the complex, and ultimately the structure and mechanism of the enzyme.

In this study, we combined *in vivo* and *in vitro* analyses of the *Synechocystis* MgCH complex. Through a combination of co-purification of native and recombinant MgCH, XL-MS and thermophoretic analysis, we show that the ChlID AAA^+^ motor interacts with the ChlH chelation subunit via a link between ChlH and ChlD. This is primarily through the C-terminal integrin I domain of ChlD; mutagenesis of key residues in the integrin I domain MIDAS motif established its importance for forming the active chelatase enzyme.

While it is known that ChlD and ChlI form a complex [8,12], the connection between ChlID and ChlH was less well defined. Previous studies have described the importance of the polyproline linker and AAA^+^ domain in ChlD to the activity of MgCH [12,15,41,42], but there has been limited description of the role of the C-terminal integrin I domain of ChlD, other than the work of Axelsson et al. [14] which revealed the importance of the MIDAS residues for chelatase activity. Our work has provided a biochemical and kinetic counterpart to the results of the recent study by Luo et al. [15], which used yeast two-hybrid analysis to show that ChlD interacts with ChlH, although our work proposes a different location for this protein–protein interaction. We have previously shown that C- and N-terminal truncations of ChlD from *Synechocystis* and *Thermosynechoccus elongatus* are not able to produce active chelatases [11,13], and that the N-terminal AAA^+^ domain of ChlD is primarily responsible for interaction with ChlI [11].

## Abbreviations

AAA*^+^*: ATPases associated with various cellular activities
*K*_d_: dissociation constant
MgCH: magnesium chelatase
MIDAS: metal ion-dependent adhesion site
MST: microscale thermophoresis
VWFA: von Willebrand factor A
XL-MS: cross-linking mass spectrometry
β-DDM: dodecyl-*β*-maltoside
