# Supplementary material for: The ChlD subunit links the motor and porphyrin binding subunits of magnesium chelatase
Source: Biochem J. 2019 Jul 2;476(13):1875–87. doi: 10.1042/BCJ20190095 (PMC6604950; doi:10.1042/BCJ20190095)
Supplement: Supplementary Tables and Figures [file BCJ-476-1875-s1.pdf]

Strain	Description	Source
WT	Glucose tolerant wild-type strain of *Synechocystis* sp. PCC 6803.	Dr Roman Sobotka, Centre Algatech, Czech Republic
FLAG-ChlD	Gene encoding 3×FLAG tagged ChlD expressed from *psbAII* promoter. Kanamycin resistant.	This study
FLAG-ChlD Δ*chlD*	Native *chlD* (slr1777) deleted from FLAG-ChlD strain. Kanamycin and zeocin resistant.	This study

S1 Table. Strains of *Synechocystis* used in this study

Primer name	Sequence (5´to 3´)
AH47	AAACGCCCTCTGTTTACCCA
AH48	TCAACCCGGTACAGAGCTTC
AH282	ATTTGGCGGCCGCAACCACCCTGACCCCCTTTATTC
AH283	ACTAAAGATCTCTATTGCATGTCGGCGATCGCCTGG
AH434	TCAATGGCATCCAGTTTGAC
AH435	ACATTAATTGCGTTGCGCTCACTGCGGAAATTGAGGGGAATAAAGG
AH436	CAACTTAATCGCCTTGCAGCACATGACCAAGGTATTGCCTCCATG
AH437	AGACCAGTCTGAGGCTCTAGTCC
AH438	CTGGGTTTCCAATTGATCCAGG

S2 Table. Primers used for generating *Synechocystis* FLAG-ChlD *ΔchlD strains* used in this study.

ChlD variant	Forward Primer (5´to 3´)	Reverse Primer (5´to 3´)
D487E	TTGTGTTTTTGGTGGAAGCGTCGGGTTCCATGG	ATGGAACCCGACGCTTCCACCAAAAACAC
S489A	TTGGTGGATGCGGCGGGTTCCATGG	AACGCCATGGAACCCGCCGCATCCACC
S491A	ATGCGTCGGGTGCCATGGCGTTGAATCG	ATTCAACGCCATGGCACCCGACGCATCC
S554R	TTGCCCTGTGGCGGTGGTCGTCCCCTTTCC	AAGCCGTGGGAAAGGGGACGACCACCGCCACAGG

S3 Table. Primers used for generating MIDAS site directed mutants of ChlD used in this study.

Gel band	Protein	-(log_{10} p-value)	No. of peptide spectrum matches	Sequence coverage (%)
Upper (*ca.* 470 kDa)	ChlH	657	547	50.6
	ChlD	456	421	57.4
Lower (*ca.* 370 kDa)	ChlH	1980	1239	80.8
	ChlD	929	745	79.7

S4 Table. Analysis by mass spectrometry of native-PAGE gel bands corresponding to putative ChlH-ChlD complexes. Bands were excised and the proteins subjected to in-gel digestion with trypsin. Peptides were extracted and analysed by nanoLC-MS/MS. Protein components were identified by database searching as described in Experimental Procedures. The p-value is the probability that the protein identification is due to random events.

Peptide in ChlH	Crosslinked site in ChlH	Peptide in ChlD	Crosslinked site in ChlD	No. of peptide spectrum matches	Best 2-D PEP
K.**E**NSSGAGFQDAMLK.L	E150	K.**K**R.L	K543	5	3.2E-7
R.L**E**AIAQR.A	E432	K.**K**R.L	K543	1	1.3E-3
K.EFGNVFIGVQPTFGY**E**GDPMR.L	E581	K.**K**R.L	K543	1	1.8E-9
R.SASPHHGFAAYYTYLNHIWKA**D**AVLHFGT HGSLEFMPGK.Q	D613	K.LVR**K**AGALIVFLVDASGSMALNR.M	K477	1	2.7E-3
R.NSDKGILA**D**VELLQDITLATR.A	D824	R.QVIVEQGDIRG**K**K.L	K472	1	5.3E-7
R.AENGGNYP**E**TIASVLWGTDNIK.T	E978	K.**K**R.L	K543	1	7.4E-4
K.TA**D**ATFQNLDSSEISLTDVSHYFDSDPTK.L	D1156	R.G**K**K.L	K472	1	1.6E-9
K.TADATFQNL**D**SSEISLTDVSHYFDSDPTK.L	D1163	K.**K**R.L	K543	1	1.0E-12
K.APAAYIA**D**TTTANAQVR.T	D1200	K.G**K**.V	K433	4	2.0E-8
K.WY**E**GMLSHGYEGVR.E	E1230	K.**K**FVSTGFGK.E	K635	2	2.7E-7
R.LVNTMGWSATAGAV**D**NWVYEDANSTFIK .D	D1261	R.QAVELVIVPRSVLMDNPPPPEQAPPPPPPP QNQDEG**K**DEQEDQQDDK.E	K353	1	4.0E-6

S5 Table. XL-MS of a putative ChlH-ChlD complex using 1-ethyl-3-(3-dimethylamnopropyl)carbodiimide-HCl (EDC). These results were generated as in S2 Table. Crosslinked residues are highlighted in bold within the peptide sequences. 2-Dimensional posterior error probability (2-D PEP) is the probability that a peptide spectrum match is a random event.

ChlD MIDAS Mutant	T_m / °C
**WT**	50
**D487E**	47
**S489A**	60
**S491A**	46
**S554R**	54

S6 Table. Melting temperatures of ChlD MIDAS Mutants. The thermal stabilities of all of the mutants are comparable to WT and well above the 34 °C used in assays. This would suggest that instability of the mutants is not the cause of the abolished or greatly reduced chelatase activity.

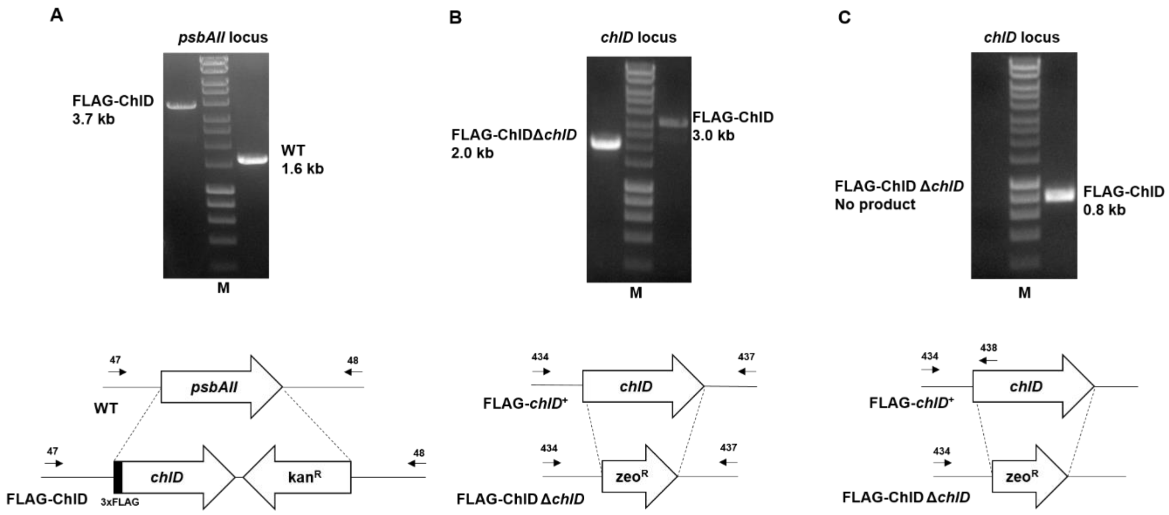

S1 Fig. Generation of *Synechocystis* strains used in this study. A Integration of gene encoding 3×FLAG-tagged ChlD at the *psbAII* (slr1311) locus in strain FLAG-ChlD. Agarose gel analysis with primers flanking the integration site shows that a single larger PCR product is amplified from the FLAG-ChlD genomic (g)DNA compared to that from the wildtype (WT) due to insertion of the tagged gene and the kanamycin resistance cassette (kan^R) in place of *psbII*. **B-C** Deletion of the native *chlD* (slr1777) gene in strain FLAG-ChlD Δ*chlD*. **B** Agarose gel analysis with primers flanking the deletion site shows that a smaller PCR product is amplified from FLAG-ChlD Δ*chlD* gDNA compared to FLAG-ChlD gDNA due to replacement of the *chlD* gene with the smaller zeocin resistance cassette (zeo^R). **C** To confirm that the FLAG-ChlD Δ*chlD* has fully segregated at the *chlD* locus PCR with a reverse primer internal to the deleted portion of the gene was performed. A product is present using FLAG-ChlD gDNA as template but absent when using gDNA from the FLAG-ChlD Δ*chlD* strain. In all panels the position of primers used in the PCR analysis are indicated with small black arrows and lane M contains Hyperladder™ I (Bioline, UK).

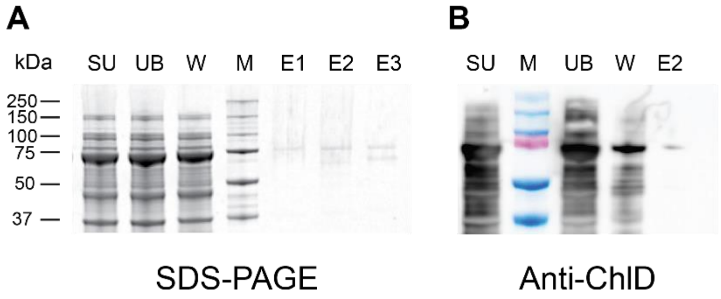

S2 Fig: Non-tagged ChlD does not purify on a StrepTrap column. (A) *Synechocystis* non-tagged ChlD was applied to a StrepTrap HP column. Lanes: SU, cell supernatant; UB, unbound fraction; M, molecular weight markers as indicated on right hand side; W, Binding buffer wash, Elutions (E1 – 3): Binding buffer with 2.5 mM biotin. **(B)** Western blot analysis of StrepII-ChlH and non-tagged ChlD fractions from **A**, showing the presence of ChlD in both the supernatant, unbound and wash fraction, but only a comparitively small amount in the elution fraction.

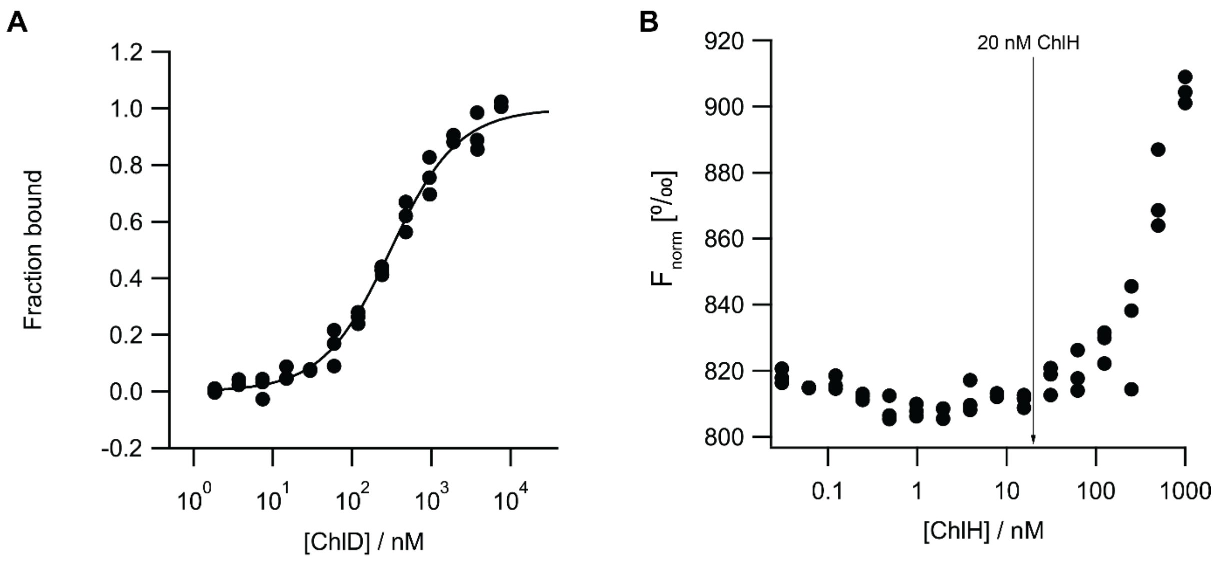

S3 Fig. Monitoring self assembly of (A) ChlD and (B) ChlH. Proteins were labelled with NHS-NT-647 and MST was conducted. (A) A single site binding model fits the data, resulting in a K_d = 296 nM, suggesting that ChlD may be binding to ChlH as a dimer. (B) As indicated by the arrow, there is no observed interaction of ChlH with itself at the concentrations used in ChlD titrations (20 nM). It does seem that ChlH will dimerise at higher concentrations, however. Thermophoresis was performed where each chelatase subunit was titrated into 20 nM labeled of the same subunit in 50 mM Tris/NaOH, 10mM Mg^{2+} 0.2% Pluronic-F127, pH 7.8.

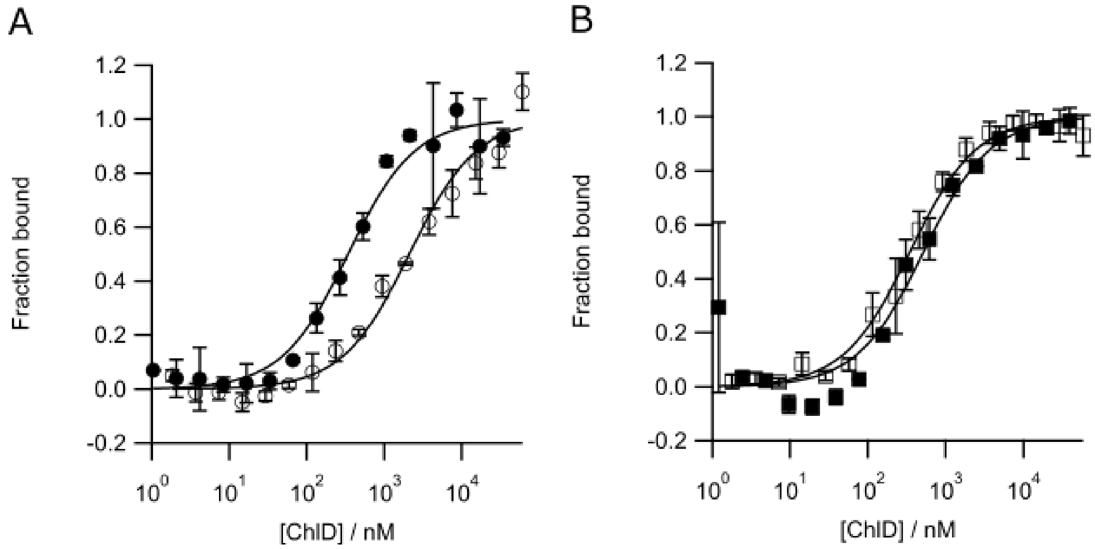

S4 Fig. The Quintuple mutant (QuinE) of ChlD does not have a Mg^{2+} dependent K_d for ChlH. Unlike WT ChlD, the interaction between ChlH and the non-cooperative QuinE mutant of ChlD is not dependent on Mg^{2+}. MST traces of WT (A) and QuinE (B) in the presence and absence of Mg^{2+} (filled and unfilled symbols respectively). The fitted curves reveal a K_d of 338.5 ± 39.7 nM and 512.5 ± 161.31 nM for the QuinE mutant in the presence and absence of Mg^{2+}, respectively.

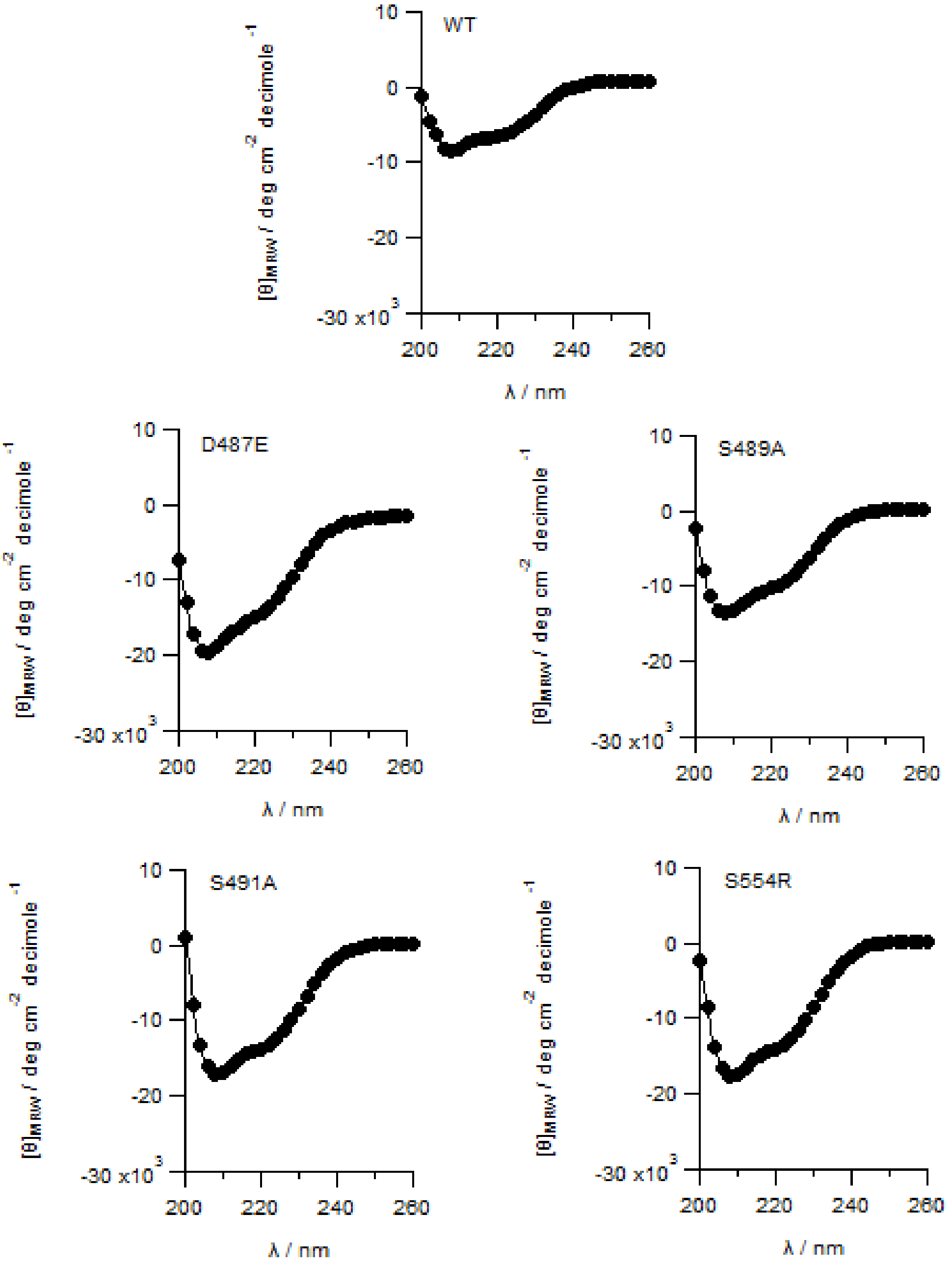

S5 Fig. CD spectra of the ChlD MIDAS mutants. All four mutants have a broadly similar CD spectra to the WT, suggesting that secondary structure of these mutants has not been disrupted. Spectra were recorded with a JASCO-810 spectrometer (JASCO, Great Dunmow, UK). Protein (0.05 mg ml^{-1}) was in a 5 mM sodium phosphate buffer, 1 mM β-mercaptoethanol, pH 7.5. Spectra were recorded from 260 to 200 nm (1 nm steps, 4 s/nm, 4 accumulations) and background subtracted.

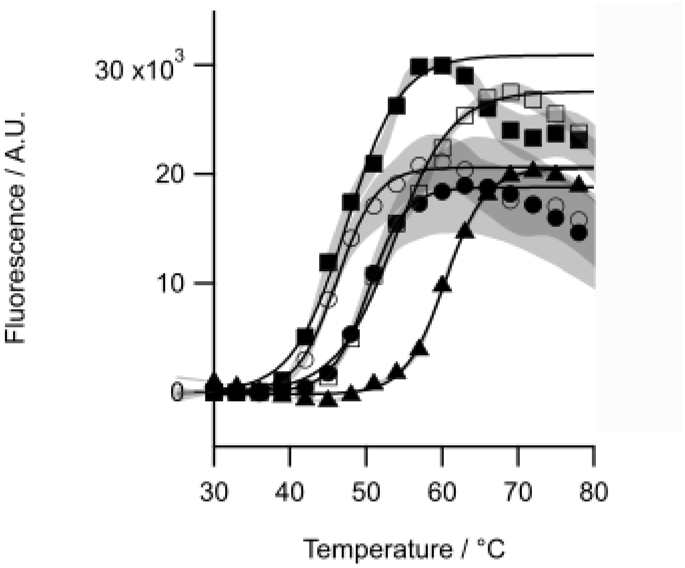

S6 Fig. Temperature stability of MIDAS mutants. All mutants (D487E, filled square; S489A, filled triangle; S491A, open circle; S554R, open square) have a thermal stability comparable or above WT (filled circles), with all calculated T_m values greater than the activity assay temperature of 34 °C. T_m values are shown in Table S4. Assay contained 5 µM ChlD, 10 mM $MgCl_2$, 1x SYPRO Orange and 1x thermal shift buffer. Temperature ramp rate was 1 °C per cycle per minute. Fluorescence was read at each temperature. Every 3 values are shown for clarity, and can be described by equation 3 to calculate T_m values.

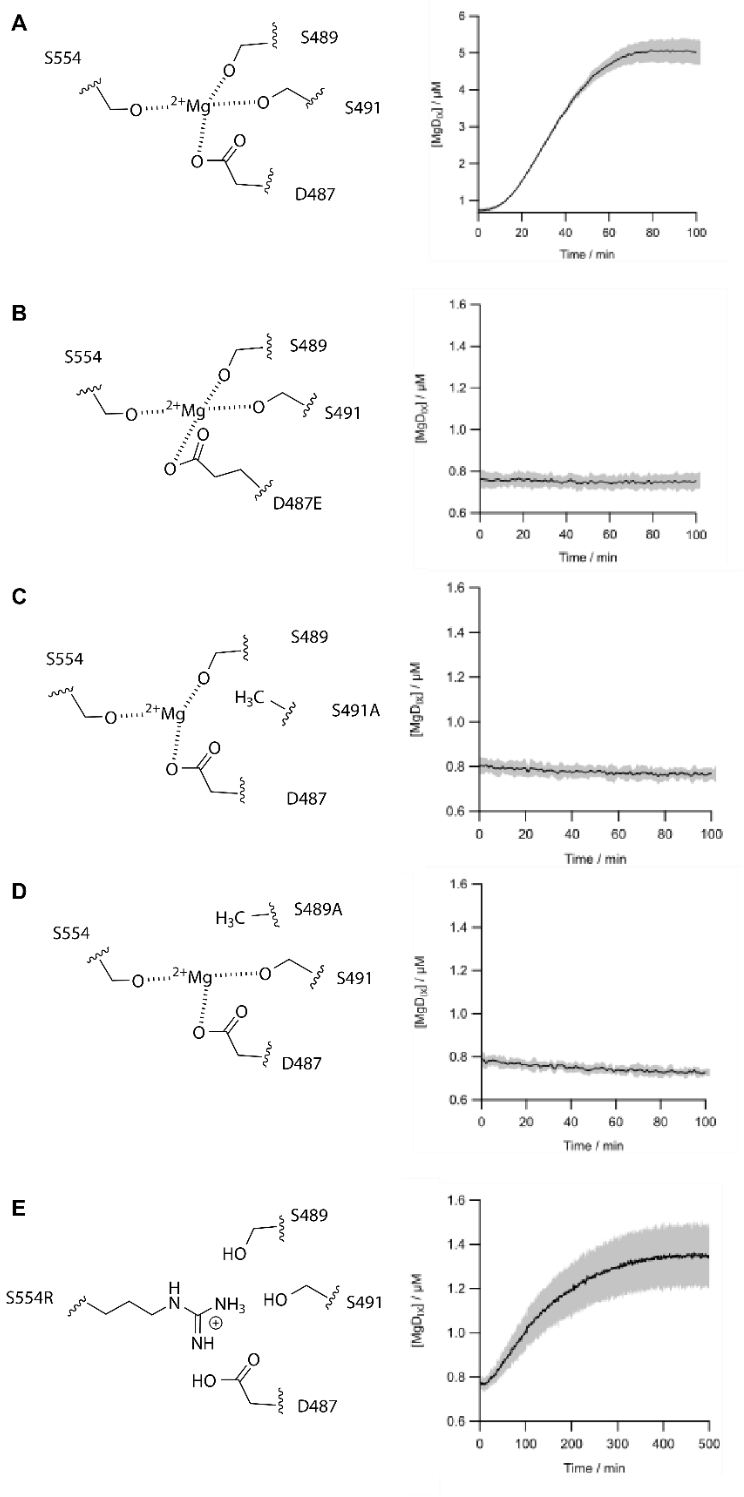

S7 Fig. Activity assays of ChlD MIDAS mutants. Schematic representation of mutant and subsequent chelatase activity of A, WT; B, D487E; C, S491A; D, S489A; and E, S554R. Assays performed under the same conditions as Fig. 3. Of the mutants, only ChlD S554R (open circles) showed any activity, although this was severely impaired when compared to WT (k_{rel} = 5 % c.f. WT ChlD).
